# RETRACTED ARTICLE: Agenda to adoption: understanding the mechanisms driving fee-free policy development in Sub-Saharan Africa through policy change frameworks

**DOI:** 10.1007/s11077-022-09473-3

**Published:** 2022-08-17

**Authors:** Gabriel Asante, György Gajduschek, Attila Bartha

**Affiliations:** 1 Doctoral School of International Relations and Political Science, Corvinus University of Budapest, Budapest, Hungary; 2 Institute of Economic and Public Policy, ELKH Centre for Social Sciences, Corvinus University of Budapest, Budapest, Hungary

Due to a publisher error, this article was mistakenly published and has been removed.

